# Building and Testing of a Conceptual Model to Describe and Measure the Health of People as Affected by Post-traumatic Stress Disorder During Social Unrest: A Confirmatory Factor Analysis and Structural Equation Modeling

**DOI:** 10.3389/fpubh.2022.838606

**Published:** 2022-03-09

**Authors:** Eva Yin-han Chung

**Affiliations:** Department of Special Education and Counseling, The Education University of Hong Kong, Hong Kong, Hong Kong SAR, China

**Keywords:** community health, participation, stigma, PTSD, social unrest

## Abstract

**Background:**

Social unrest affects people's health and well-being. People's health-related needs during social unrest are concerns in both research and clinical practice. This study aimed to build and test a framework to describe and understand the health status and needs of people with post-traumatic stress disorder (PTSD) during social unrest.

**Methods:**

This study was a cross-sectional survey. A total of 460 people who had experienced post-traumatic distress as a result of the social unrest in 2019 and 2020 were included. A conceptual model comprised four essential areas, namely posttraumatic distress symptoms, participation restrictions, perceived stigma and functional disability, was built from literature. Part 1 validated four instruments that evaluate and define the factor structure of these four areas, In Part II, structural equation modeling was used to test and validate a combined model.

**Results:**

Factors underlying the four areas were defined. Analysis using structural equation modeling confirmed a best fit of the model. PTSD symptoms, perceived stigma and participation restriction during social unrest contributed significantly to functional disability; PTSD symptoms exerted a direct effect on participation restriction and perceived stigma; and the effect of PTSD symptoms on functional disability was mediated through its influence on perceived stigma during social unrest.

**Conclusions:**

A community-based inclusive approach is essential to understand the holistic needs of people with PTSD during social unrest. To improve health and well-being in addition to evaluating mental health impacts, considering interactions with the rapid change and stressful social environment is essential.

## Background

Social unrest affects people's health and well-being. Health encompasses physical, mental, and social well-being (1) and is not merely an absence of disease or infirmity (2). According to the International Classification of Functioning, Disability and Health (ICF), health and functioning is a dynamic interaction between health conditions and contextual factors, both personal and environmental. The health of people during social unrest cannot be fully understood unless its impacts on physical and mental health and social participation are determined and defined.

The health-related problems of people during social unrest are recent concerns of both research and practice. In June 2019, extensive protests began in Hong Kong, bringing with them prolonged social unrest that has extended into 2020. A research study on this unrest reported weighted prevalence rates of depressive symptoms and suicidal ideation of 37.4 and 4.3%, respectively (3). A prospective study revealed a high prevalence of probable depression and suspected posttraumatic stress disorder (PTSD) during this period of social unrest (3). PTSD is categorized in DSM 5 as a traumatic and stress-related disorder which requires a direct or indirect exposure to a traumatic event. Clusters of symptoms include intrusion, avoidance, alterations in cognition and mood, and alterations in arousal and reactivity (4). However, studies have tended to focus on the impacts of social unrest on mental health rather than the full spectrum of health-related issues (5, 6). PTSD may associate with an increased functional disability because such reduction in function is seen across a broad set of functional domains that include aspects of physical and mental health (7).

Health of people during social unrest has long been studied under a medical model. Health issues is understood as an individual and a medical phenomenon that results in limited psychological and mental functioning (8). Post-traumatic stress disorder and depression are the two commonly reported health issues of people under social unrest (5, 9). However, health is more than the evaluation and understanding of the medical symptoms. It is essential to look into the impact of social unrest on people's health in the areas of activity and social participation (10). This study advocated for a thorough understanding of people's health during social unrest in relation to its social context. It should be able to reflect the impact of the rapid change in individuals, communities, and social contexts during social unrest (11).

### Formulation of a Conceptual Framework

This study aimed to build a framework to understand the health of people with PTSD during social unrest based on a community-based inclusive development approach. This approach encourages the building of inclusive, resilient, and equitable communities in which disadvantaged people are empowered and given the opportunity to lead inclusive and healthy lives (12). The delineation of the conceptual framework for the current study was built upon the ICF (10). Essential domains of health in relation to PTSD as conceptualized using a community-based inclusive development approach were delineated based on the literature (3, 9, 13–19).

#### Domain 1: Psychological Impairments Due to Social and Political Unrest

The most commonly reported psychological impairment among people during social unrest is post-traumatic distress (3). Trauma during social unrest can be viewed from a politico-psychological perspective. Such perspective highlights the interaction between the political context and the mental conditions of trauma-exposed individuals (9). Posttraumatic distress arises from experiences of trauma, such as combat, witnessing a violent act, or sustaining a debilitating injury. Symptoms of posttraumatic stress include reexperiencing the trauma through intrusive, distressing recollections of the event, flashbacks, and nightmares; hyperarousal; and emotional numbness and avoidance of reminders (places, people, and activities) of the trauma (13).

#### Domain 2: Functional Disability-Limitations in Body Functions and Daily Activities

Impairment of body structures and functions represents a deviation from certain generally accepted population standards. Such impairments can be temporary or permanent; progressive, regressive, or static; and intermittent or continuous (15). Activities include everything from basic activities such as self-care to composite areas such as interpersonal communication or employment (15). Activity limitations are difficulties an individual may face in executing these activities. The core set of activity limitations among people with PTSD includes solving problems, carrying out daily routines, handling stress, having conversations, using transportation, looking after one's health, acquiring goods and services, doing housework, assisting others, having interpersonal interactions, engaging in social and family relationships, and engaging in education or work (16).

#### Domain 3: Restrictions in Social Participation

Participation is involvement in life situation (15). Participation has been variously defined in terms of individual, personalized relationships, through broad collective citizen involvement, meaningful engagement, active involvement in policy implementation, shared or delegated power, and coproduction (17). Activities and participation encompass the full range of life areas. Participation restrictions are problems an individual may experience in involvement in life situations and fulfillment of life roles. Limitations and restrictions should be assessed from the perspective of the person involved and against a generally accepted population standard. Measurements and records of such limitations and restrictions document the discordance between the observed and expected performance as well as between the majority and the disadvantaged minority, from a person-centered perspective.

#### Domain 4: Contextual Factors and Social Stigma Associated With Social and Political Unrest

Contextual factors represent the complete background of an individual's life and living. Discrimination and social stigma are critical environmental factors that limit participation and contribute to disability (14). Stigma is defined as a set of prejudices, stereotypes, discriminatory beliefs, and biases linked to the characteristics that differentiate a person from others (18). Perception of stigma and experience of discrimination induce feelings of shame and may also cause anxiety, depression, and isolation (14). Regardless of their political stance, people may wish to hide their PTSD symptoms from their families or employers to avoid social stigma. Stigmatized identities carry different levels of social devaluation, which can be culturally constructed (19).

As concluded from the above review, a preliminary model with four major areas were formed to understand the health of people during social unrest: (1) posttraumatic stress and psychological impairments, (2) body functions and activity limitations (disability), (3) participation restrictions, and (4) perceived stigma.

### Research Aims and Objectives

The aim of this study was to formulate and test a proposed model to understand and predict the health and disability of people with PTSD during social unrest. The research questions are as follows:

1. What are the valid measurements of PTSD symptoms, participation restrictions, perceived stigma and functional disability for people affected by post-traumatic distress during social unrest?
2. What are the factor structure of the four essential constructs as confirmed with evidence?
3. What is the model of best fit to measure and predict the disability of people with PTSD during social unrest?

This study is important in understanding the factors for health and disability among the people with PTSD in social unrest. There is currently no universal framework to understand and describe the health and disability of people with PTSD based on a community-based inclusive approach. Health and inclusion cannot be fully understood by merely measuring the ability of an individual in relation to the medical condition. The World Health Organization (20) states the importance of placing disability issues and people with disabilities in the mainstream of activities and removal of all discriminating barriers to enhance inclusion. According to the International Classification of Functioning, Disabilities and Health (15), social and physical environmental factors do affect participation and inclusion. Therefore, it is essential to have a valid and comprehensive model to understand the essential factors affecting the health and quality of inclusion among the people with PTSD during social unrest.

## Methods

### Research Design

It was a cross-sectional study. The study was conducted following three steps.

#### Step 1. Validating the Measurements

Based on the aforementioned conceptual base to understand the impact of social unrest on the health on people, the four essential areas to understand the health and disability of people with PTSD were regarded as (1) PTSD symptoms, (2) social stigma, (3) participation, and (4) functional disability. A valid and reliable measurement tool was required to measure each area. Four instruments were then selected based on their psychometric properties and relevance as reviewed by literature. The four selected instruments were: the (1) PTSD Checklist for *Diagnostic and Statistical Manual of Mental Disorders, Fifth Edition* (PCL-5), (2) Explanatory Model Interview Catalog (EMIC) Stigma Scale, (3) Participation Scale (P-scale), and (4) WHO Disability Assessment Schedule 2.0 (WHODAS 2.0). Reliability analysis and Rasch model analysis were done to confirm the reliability and dimensionality of these scales for use with people with PTSD.

#### Step 2. Confirming the Factor Structure Using a Confirmatory Factor Analysis

Constructs of each domains must be defined by measureable variables that can be collated to form a factor (21). Each factor represents a unique combination of items that reflects a different theoretical component of the construct. This part focused on confirming the constructs of the proposed model. Confirmatory factor analysis was done to confirm the factor structure.

#### Step 3. Testing of a Combined Model Using Structural Equation Modeling to Predict Functional Disability of People With PTSD During Social Unrest

**Figure 1:**
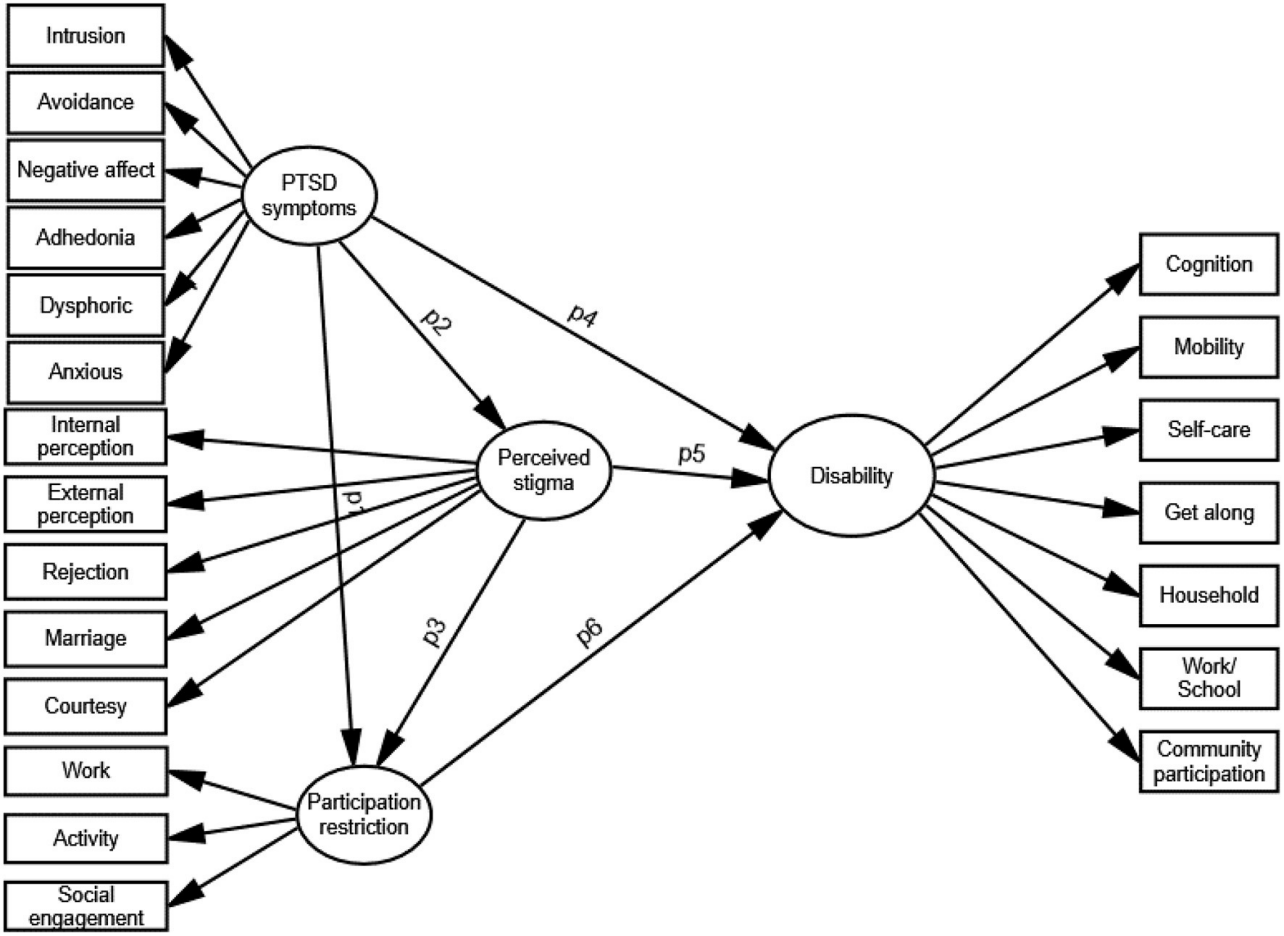
The proposed model for path analysis.

Structural equation modeling (SEM) was used to test the relationship among PTSD symptoms, participation restrictions, stigma and functional disability. Three hypotheses were tested through this model. The path diagram for testing is showed in Figure 1.

H1: Perceived stigma is predicted ty PTSD symptoms among people affected by posttraumatic distress (p2).
H2: Participation restriction is predicted by PTSD symptoms (p1) and perceived stigma (p3) among people with PTSD.
H3: Functional disability is predicted by PTSD symptoms (p4), perceived stigma (p5) and participation restriction (p6) among people with PTSD.

### Participants

Target participants in Steps 1 and 2 were the people affected by post-traumatic distress in the social unrest; while participants in Step 3 were those matching the diagnostic criteria of PTSD. In Step 1 of this study, people affected by post-traumatic distress in the social unrest in Hong Kong were recruited. The inclusion criteria were (1) being aged 18 years and over, (2) reported experience of post-traumatic distress in social unrest, (3) having mental clarity, and (4) having sufficient cognitive ability to comply with the instructions to complete the tests. Participants were recruited openly through different sources. Different organizations working with people with PTSD in local community were contacted and liaised by the research team, with the research team liaison reaching out to the target groups and participants. A total of 460 participants were enrolled into this study. Step 2 (validation of instruments) involved all enrolled participants (*n* = 460) while Step 3 (SEM) involved only those who matched with the diagnostic criteria as stated in the DSM 5 as revealed by the PCL 5. Participants got a total score higher than 31 were eligible for this part of the study (22). A total of 260 participant met this criterion and were included. Ethical approval was obtained from the ethics committee of the university (202020210406). Written informed consent was obtained from all participants.

### Data Collection

Data was collected through online questionnaire. The complete set of self-administered questionnaires as well as written instructions and completion guides were electronically distributed to all participants with their consent.

### Instruments

The Posttraumatic Stress Disorder Checklist for the DSM-5 (PCL-5) was used in this study to evaluate the level of posttraumatic distress symptoms. It is a 20-item self-report measure that assesses PTSD symptoms. Respondents indicated the extent to which they had been bothered by each PTSD symptom over the past month on a 5-point scale, with responses ranging from 1 (*not at all*) to 5 (*extremely*). The PCL-5 corresponds to DSM-5 criteria for PTSD. In its original language, it has strong internal consistency, test–retest reliability, and convergent and discriminant validity (23).

The EMIC Stigma Scale is a 15-item instrument that was designed to measure perceived stigma among the people with disabilities (21). This scale was used in this study to measure the painful inner struggle linked to PTSD with regard to equal opportunities to live and participate in local community. There is currently no stigma scale for use by people with PTSD. Each question has four options: “yes”, “possibly”, “uncertain”, and “no”. It employs a reverse scoring method on a 4-point scale. A higher score implied a higher level of perceived stigma faced by the respondent.

The P-Scale is an 18-item interview-based instrument that measures social participation. It covers the major life areas: work/school (learning and applying knowledge, general tasks and demands), community life (communication, mobility, self-care, domestic life), and social life (interpersonal interactions and relationships, civil life) (21, 24). Respondents are ranked by five levels of participation. A higher total score represents more participation restrictions and thus less general participation.

The WHO Disability Assessment Schedule 2.0 (WHODAS 2.0) measures the level of functioning and disability in six domains of life: cognition, mobility, self-care, getting along, life activities, and participation. WHODAS 2.0 is a practical, generic assessment instrument that can measure disability at the population and clinical levels (25). It contains 36 questions related to the functioning difficulties experienced by respondents in the six life domains over the previous 30 days. The total score of WHODAS ranges from 0 to 100. A higher score indicates a high level of disability.

### Data Analysis

#### Validation of the Instruments

The data were cleaned to exclude missing data. The internal consistency and construct validity, of these scales as applied to these specific participants were examined. Reliability analysis of the four instruments was performed using IBM SPSS Statistics for Windows, version 25 (IBM Corp., Armonk, NY, USA). Cronbach's alpha was calculated to assess the internal consistency of the instruments. Convergent validity and reliability analysis of the four scales was conducted using SPSS. Rasch model analysis of the four scales was performed using Winstep to examine the internal validity and dimensionality of the overall score of the four scales. Data were first cleaned based on diagnosis of misfit person. Participants were excluded if the point measure correlation had a negative value, the outfit mean-square (MNSQ) value was >2, and the Z-standard value was >2 (26). Dimensionality is a key part of the assessment of construct validity because it indicates whether the items of the individual scale measure a single underlying dimension (21, 27). To verify that the scale is unidimensional, the observed variance explained by the measures should roughly match the expected variance in the model (28), and the unexplained variance in the first contrast should be smaller than the raw variance explained by the items (29).

#### Confirming the Factor Structure Using Confirmatory Factor Analysis

Confirmatory factor analysis (CFA) was conducted for each instrument to determine their factor structure using SPSS Amos Version 26 (IBM Corp.). The factor structure of each instrument for testing was mostly based on literature data. The estimated correlation among factors and model fit indices were used to assess the model fit. Factor loadings of constructs >0.70, 0.63, 0.55, 0.45, and 0.32, were considered excellent, very good, good, fair, and poor, respectively (30). To ensure acceptable fit, a model should have root mean square error of approximation (RMSEA) indices, comparative fit index (CFI) values, goodness of fit index (GFI) values, and chi-square/degrees of freedom (CMIN/df) ratios close to 0.06, 0.9, 0.9, and <3, respectively (31).

#### Testing of a Combined Model Using Structural Equation Modeling to Predict Functional Disability of People With PTSD

The results from the CFA provided a plausible set of health-related facets as well as a hierarchical model describing their arrangement in each variable. Independent variables were PTSD symptoms, participation restrictions and perceived stigma, while the dependent variable was level of disability (Figure 1). SEM analysis was conducted using SPSS Amos Version 26 (IBM Corp). The RMSEA, CFI, GFI, and CMIN/df were used to assess model fit. To test which statistic fit best, comparing how well the proposed model fit with the empirical data was essential. Covariance among PTSD symptoms, perceived stigma and social participation; and regression weight of the predictors toward the outcomes (disability) were computed. *Post-hoc* power analysis was conducted using semPower to determine the actually achieved power to detect model misspecification and target effect (32).

To test which statistic fit best, comparing how well the proposed model fit with the empirical data was essential. Covariance among PTSD symptoms, perceived stigma and social participation; and regression weight of the predictors toward the outcomes (disability) were computed.

## Results

### Validation of the Instruments

#### Reliability

The internal consistency of the four instruments was high. Cronbach's alphas for the PCL 5, WHODAS 2.0, P-scale, and EMIC stigma scale were 0.94, 0.97, 0.92, and 0.80, respectively. The Cronbach's alpha values of all items in each scale were >0.75, indicating that the overall reliability of the scale was not greatly affected by any one item (33).

#### Convergent Validity

Correlations among the total scores of the four scales were evaluated to test the convergent validity and assess the relationships between the four scales. Because the total scores of all four scales were not normally distributed, Spearman's rank-order correlation was used. As presented in Table 1, all comparisons revealed significant correlations, confirming the convergent validity of the four scales.

**Table 1:** Correlations across the four scales.

	**PCL 5**	**P-scale**	**WHODAS**	**EMIC**
PCL 5	1.000	0.511**	0.720**	0.430**
*P*-scale	0.511**	1.000	0.570**	0.381**
WHODAS	0.720**	0.570**	1.000	0.468**
EMIC	0.430**	0.381**	0.468**	1.000

** *Correlation is significant at the 0.01 level (2-tailed)*.

#### Rasch Model Analysis

The internal validity and dimensionality of the four scales for use with people experienced post-traumatic distress in Hong Kong was confirmed using Rasch model analysis. After data cleaning, 455, 442, 432, and 434 valid cases were included in the analysis of the PCL 5, WHODAS 2.0, P-scale, and EMIC stigma scale, respectively. Determined from the summary statistics of the Rasch model analysis on the four scales (Table 2), all scales had good reliability of the scale and high replicability of person ordering. The commonly accepted range for the MNSQ value is 0.6 to 1.4, and that for the ZSTD is −2 to +2 (34). As presented in Table 3, the infit and outfit MNSQ and ZSTD values were within the acceptable range. The results of the analysis indicated that the person fit and item fit of all scales were good. Rasch-residual-based principal component analysis (PCAR) indicated that the unexplained variance explained by the first contrast in the PCL 5, WHODAS 2.0, P-scale, and EMIC stigma scale was less than the variance explained by the items. In all four scales, the observed variance explained by the measure was similar to the expected variance in the model (Table 3).

**Table 2:** Summary statistics of the four scales using Rasch model analysis.

	**Item reliability**	**Person reliability**	**Person**				**Item**			
			**MNSQ**		**ZSTD**		**MNSQ**		**ZSTD**	
			**Infit**	**Outfit**	**Infit**	**Outfit**	**Infit**	**Outfit**	**Infit**	**Outfit**
PCL 5	0.99	0.92	1.01	1.01	−0.2	−0.1	1.02	1.01	0.0	−0.3
WHODAS 2.0	0.99	0.94	1.03	1.03	−0.1	−0.1	1.04	1.03	0.2	0.1
*P*-scale	0.97	0.81	1.08	1.06	−0.2	−0.2	1.02	1.07	0.1	0.5
EMIC	0.98	0.73	1.04	1.06	0.0	0.0	1.02	1.07	−0.3	0.2

**Table 3:** Rasch-residual-based principal component analysis.

	**Observed variance explained by the measure**	**Expected variance of the model**	**Unexplained variance explained by 1st contrast**	**Variance explained by the items**
PCL 5	54.80%	54.90%	5.60% (2.5)	31%
WHODAS 2.0	52.70%	53.40%	4.90% (3.7)	33.80%
*P*-scale	39.30%	40.90%	6.60% (2.0)	29.60%
EMIC	30.00%	30.20%	10.00% (2.1)	23.90%

### Confirming the Factor Structure Using Confirmatory Factor Analysis

#### PTSD Symptoms as Measured by the PCL5

After data cleaning, 460 participants remained for analysis. All items were normally distributed, with univariate skewness and kurtosis values lower than 2 and 7, respectively (35). Maximum likelihood estimation was then used to test the model. The model of six factors—intrusion, avoidance, negative affect, anhedonia, dysphoric arousal, and anxious arousal—as proposed by Liu et al. (36), was initially tested using the participant data. Factor loadings of all items to the six factors were high (0.44–0.93), and all loadings were significant (*p* < 0.05). PTSD was a higher-order factor, and the factor loadings of the six factors to PTSD were also high (0.70–0.96). The model demonstrated a good fit to the data [χ^2^(164) = 489.965, *p* < 0.01, CMIN/df = 2.99, CFI = 0.94, GFI = 0.90, non-normed fit index (NNFI) = 0.91, RMSEA = 0.06, standardized root mean residual (SRMR) = 0.06, TFI = 0.93]. As a result, the underlying factors for PTSD symptoms as measured by the PCL 5 were defined as intrusion, avoidance, negative affect, anhedonia, dysphoric arousal, and anxious arousal.

#### Social Participation as Measured by the Participation Scale

In total, 452 participants were included for analysis. The value of Cronbach's alpha representing the internal consistency of the scale was 0.92. The data did not have a multivariate normal distribution; the critical ratio of multivariate kurtosis was 74.01 (37). Model testing was conducted through maximum likelihood estimation using the Bollen–Stine bootstrapping method (*n* = 500, with a 95% bias-corrected interval). The tested model structure was based upon our previous work on the validation of the P-Scale (21) which followed a three-factor model: work or school, community life, and social engagement. This model demonstrated an adequate fit to the data [χ^2^(132) = 475.51, *p* < 0.01, CMIN/df = 3.60, CFI = 0.91, GFI = 0.90, NNFI = 0.87, RMSEA = 0.07, SRMR = 0.09, TLI = 0.89]. Factor loadings of all items to their respective factors were high (0.61–0.76) and the three factors loaded significantly to the higher-order factors (0.74–0.98, *p* < 0.05). The three factors underlying participation were confirmed as work (or school), community life, and social engagement.

#### Perceived Stigma as Measured by the EMIC Stigma Scale

A total of 451 participants were included for analysis. The value of Cronbach's alpha was 0.79. The data did not have a multivariate normal distribution; it had a critical ratio of multivariate kurtosis of 16.87. The model comprised five factors: (1) internal perceptions, (2) external perceptions, (3) rejection, (4) marriage, and (5) courtesy stigma (38). Correlations among the five factors (correlation coefficient: *r* = 0.29–0.80) indicated no problems with multicollinearity. The model demonstrated adequate fit to the data [χ^2^(80) = 190.78, *p* < 0.01, CFI = 0.92, GFI = 0.95, NNFI = 0.88, RMSEA = 0.06, SRMR = 0.05, TLI = 0.90]. However, the factor loadings of two items to the factors were below 0.3. The theoretical base of the EMIC Stigma Scale was reviewed, and the factor Internal Perception comprised disclosure and feelings. A second-order factor model was then proposed and tested. Fit indices indicated a good fit [χ^2^(83) = 157.24, *p* < 0.01, CMIN/df = 1.89, CFI = 0.95, GFI = 0.96, NNFI = 0.90, RMSEA = 0.05, SRMR = 0.05, TLI = 0.94]. Factor loadings of the items to the factors were high (0.40–0.96), and all factors loaded significantly to the higher-order factors (0.52–0.98, *p* < 0.05). As a result, the confirmed factors underlying stigma were confirmed as internal perception, external perceptions, rejections, marriage, and courtesy stigma.

#### Functional Disability as Measured by the WHODAS 2.0

A total of 458 participants were included for analysis. Reliability analysis showed the value of Cronbach's alpha of the WHODAS 2.0 for use with people affected by post-traumatic distress in Hong Kong was 0.96.The data did not have a multivariate normal distribution; the critical ratio of multivariate kurtosis was > 5 (37). Model testing was performed through maximum likelihood estimation using the Bollen–Stine bootstrapping method. Bootstrap samples were determined at 500 samples with a 95% bias-corrected interval (39). Correlations among the variables were below 0.8 (0.36–0.78); hence, multicollinearity was not a problem. Two models were tested. The first model was 6-factor model, in accordance with the six WHODAS 2.0 domains. The second model was based on the 7-factor model, the construct validity of which Garin et al. tested and confirmed (40). This model essentially followed the six WHODAS 2.0 domains, but the domain of life activities were divided into two factors: household and work (or school) activities. In the present study, the first model had poor fit to the data [χ^2^(579) = 3337.79, *p* < 0.01, CMIN/df = 5.77, CFI = 0.78, GFI = 0.65, NNFI = 0.75, RMSEA = 0.10, SRMR = 0.11, TLI = 0.77]. The 7-factor model demonstrated a moderate fit [χ^2^(584) = 1743.65, *p* < 0.01, CMIN/df=3.00, CFI = 0.91, NNFI = 0.87, RMSEA = 0.066, SRMR = 0.069, TLI = 0.91]. Factor loadings of all items to their respective factors were high (0.53–0.93). All seven factors loaded significantly to limitations in body functions (0.58–0.91, *p* < 0.05). As a result, the seven confirmed factors underlying body functions and activities were cognition, mobility, self-care, getting along, work/school, involvement in society, and household activities.

### Testing of a Combined Model Using Structural Equation Modeling to Predict Functional Disability of People With PTSD

Two hundred sixty participants, who scored 31 or above in the PCL 5, were included in this part. The results of *post hoc* power analysis using semPower (41) showed, a sample size of 260 is associated with a power larger than 99.99% (1-beta = 0.999, degree of freedom = 181, alpha = 0.05) to reject a wrong model. The 3-factor, high order model hypothesizing the directional relationship successfully converged in 10 iterations. Using the maximum likelihood estimation, evidence from the model suggested the data did not fit the model expected [χ^2^(183) = 615.07, *p* < 0.01, CMIN/df = 3.36, CFI = 0.92, GFI = 0.88, RMSEA = 0.07, SRMR = 0.05, TLI = 0.91]. Modification indices were checked to improve the model fit. When the two errors were covaried, the model was reanalysed. The revised model fit the data well [χ^2^(181) = 485.57, *p* < 0.01, CMIN/df = 2.68, CFI = 0.95, GFI = 0.91, RMSEA = 0.06, SRMR = 0.047, TLI = 0.94].

**Figure 2:**
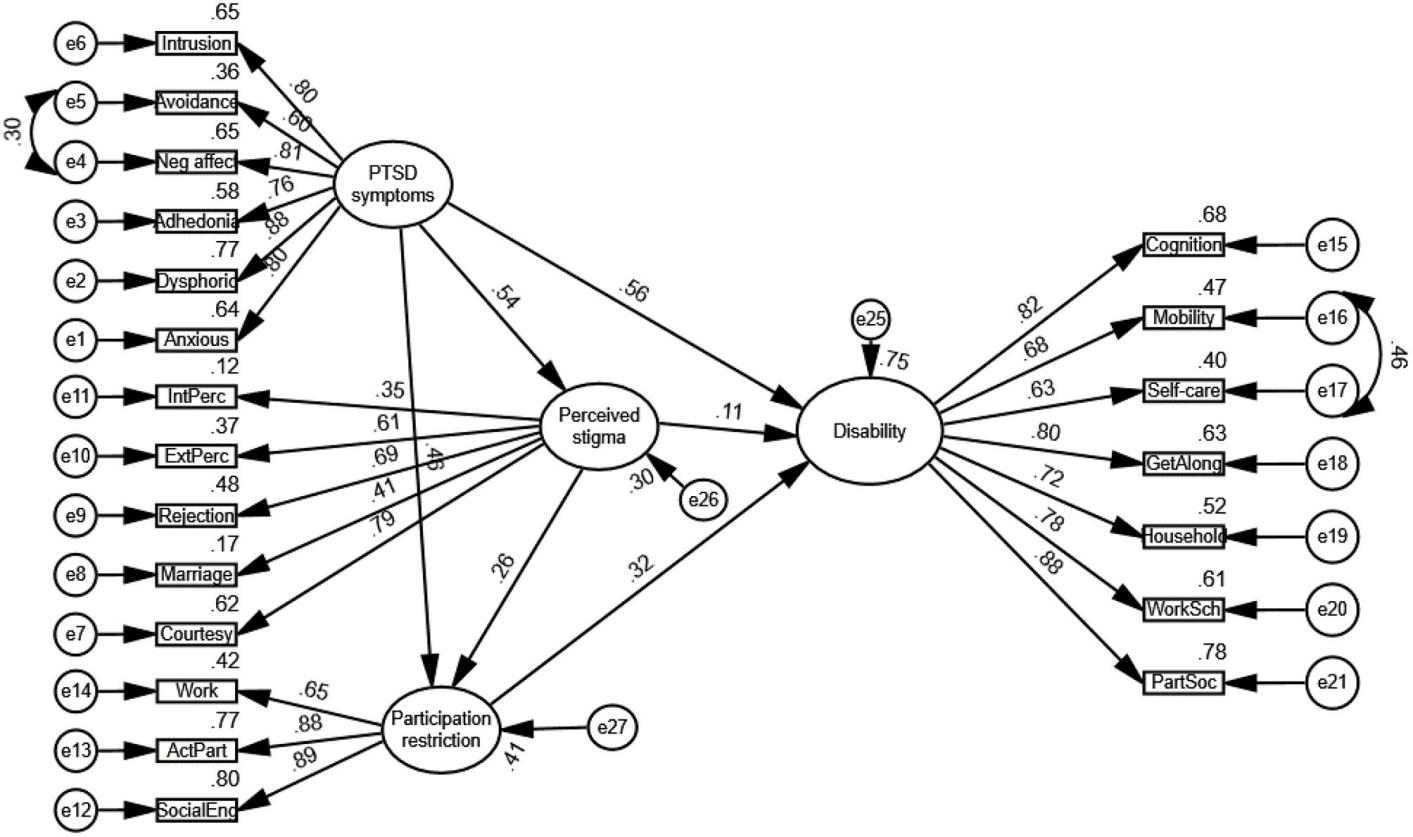
The final model.

Figure 2 indicates standardized estimates for path coefficient. Standardized estimates allow the relationships among latent variables to be compared. Figure 2 indicates a stronger relationship between PTSD symptoms and participation restriction (β = 0.46) than between perceived stigma and participation (β = 0.26). Among the three factors affecting functional disability, the strongest relationship is demonstrated between PTSD symptoms and functional disability (β = 0.56). Among the other two factors, it indicates a stronger relationship between participation restriction and disability (β = 0.32) than between perceived stigma and disability (β = 0.11).

Table 4 shows the direct and indirect and total effects utilizing structural equation modeling with bootstrapping to verify effect significance. PTSD symptoms directly affected perceived stigma, participation restriction and functional disability (*p* < 0.05). Perceived stigma had direct effect on participation restriction and functional disability (*p* < 0.05). Participation restriction directly affected functional disability (*p* < 0.05). PTSD symptoms, as the sole predictor of perceived stigma, explained 91% of the variance in perceived stigma. The relationship between PTSD symptoms and participation restriction is partially mediated by perceived stigma. Altogether, PTSD symptoms and perceived stigma explained 83% of the variance in participation restriction. Moreover, the interaction of the PTSD symptoms, perceived stigma and participation restriction explained a total of 44% of the variance in functional disability.

**Table 4:** Effects of exogenous variables on the endogenous variables.

**Endogenous variables**	**Exogenous variables**	**Direct effects**	**Indirect effects**	**Total effects**	***p***	**SMC (%)**	**Explained variance**
Stigma	PTSD symptoms	0.544		0.544	<0.001	0.296	0.91
Participation restriction	PTSD symptoms	0.459	0.142	0.601	<0.001	0.410	0.83
	Perceived stigma	0.262		0.262	<0.001		
Functional disability	PTSD symptoms	0.560	0.253	0.813	<0.001	0.749	0.44
	Perceived stigma	0.107	0.085	0.192	0.018		
	Participation restriction	0.325		0.325	<0.001		

## Discussion

### Validation of Instruments Measuring the Health and Inclusion of People With PTSD

In this study, four valid measurement tools were validated to measure the health of people with PTSD as affected by social unrest. Based on a community-based inclusive development approach, these four basic areas were regarded as essential: PTSD symptoms, participation restrictions, perceived stigma and functional disability. The PCL-5, WHODAS 2.0, P- Scale, and EMIC Stigma Scale, for which construct validity and internal consistency were confirmed in this study, can be used as screening tools to facilitate the identification of health-related needs of people affected by posttraumatic distress during social unrest. The internal consistency of these four scales was found to be high (Cronbach's α > 0.8) and it indicated a good reliability. This result was similar to the previous studies on other target groups of people with chronic disabilities (21, 23, 42).

### Confirming the Elements and Factors Under the Four Essential Areas of Concern in Community-Based Inclusive Development

The factor structures of the four instruments as confirmed with the CFA were similar to past studies when these instruments were used with other target groups. A six-factor model in PCL5 was confirmed in this study and the factor structure as revealed by the CFA was similar to Liu et al.'s study (36). The six factors of the PTSD symptoms as measured by the PCL 5 were confirmed in this study as intrusion, avoidance, negative affect, anhedonia, dysphoric arousal, and anxious arousal. In measuring participation restrictions using the P-scale, a three-factor model was confirmed. The three factors were confirmed as work/school, community life and social engagement. This result was similar to a previous study when the P-Scale was applied in the people with physical disabilities (21). The EMIC Stigma Scale was confirmed to be similar to the literature (38) and it revealed a five-factor model: internal perception, external perception, rejection, marriage, and courtesy stigma. However, it was found it this study the factor Internal Perception comprised disclosure and feelings, and therefore a second-order factor model for the EMIC Stigma Scale was confirmed. For the WHODAS 2.0, it was found the data fit better with a seven-factor model (40) rather than a six-factor model as proposed by the WHO (25). As similar to Garin et al.'s study (40), the confirmed factors were: cognition, mobility, self-care, getting along, work/school, involvement in society, and household activities.

Essential factors underlying the four areas laid a solid theoretical base for the framework. Factor loadings for the factors under each domain were high. Intrusion, avoidance, negative affect, anhedonia, dysphoric arousal, and anxious arousal were the essential factors contributing to PTSD in response to traumatic experience. People commonly report sleep disturbance, flashbacks, emotional responses, and difficulty concentrating after witnessing traumatic events, whether in person or through media sources. Cognition, mobility, self-care, getting along, household activities, work or school activities, and participation in school are limitations in body functions and activities that may lead to functional disability. Those affected may be preoccupied with thoughts related to the trauma, encounter difficulties in interpersonal relationships, or reduce their involvement in different social spheres. Participation restrictions reflect problems in the community, social life and work, or school life. Inability to fulfill life roles due to the issues may negatively affect health and quality of life. In the perceived stigma dimension, factors affecting health during social unrest were fear of disclosure, negative feelings toward oneself, rejection, courtesy stigma, problems in marriage, and negative external perceptions. All these can be disabling to one's self-image and incite negative feelings toward oneself.

### A Community-Based Inclusive Model for People With PTSD

The theoretical model of this study was confirmed using structural equation modeling. Functional disability of people with PTSD in social unrest was revealed to be predicted by PTSD symptoms, participation restriction and perceived stigma. Following the ICF framework (15), health condition, stigma and participation restriction contribute crucially to disability and functioning. In this study, PTSD symptoms, perceived stigma and participation restriction contributed significantly to functional disability during social unrest. PTSD symptoms can be disabling because traumatic experience can cause a period of tension involving intensified feelings and polarized perspectives and people strive to maintain both internal and external equilibrium during social unrest (43). Moreover, it can be a primary source of stigma that caused stress, health problems and disability. The findings of this study also suggested PTSD symptoms exerted direct effect on perceived stigma; and PTSD symptoms in relation to perceived stigma caused functional disability. People affected by posttraumatic stress during social unrest concern about receiving a label of mental illness and it can be one of the factors that impeded help seeking in early phase of PTSD that lead to functional disability (44). PTSD symptoms was found to have direct effect on participation restriction. Posttraumatic cognitions and poor trauma coping self-efficacy may hinder social participation (45). Participation in life and social activities could be limited by negative appraisals about the self and the world during social unrest. The process could also be compounded by the negative belief regarding the perception of control in the social and life situations.

### Implications on Practice

The confirmed measurement model has the potential to be used to describe and evaluate the health impact of social unrest on individuals to facilitate interventions. Past studies have focused primarily on the psychological impact of posttraumatic distress on mental health. Posttraumatic distress is a signature feature associated with traumatic experience, violence and human rights abuse (46). Other than the elevated stress level that affect the mental health of a person, this study emphasized that posttraumatic distress affected also the physical health, daily activities and social participation of a person.

To our knowledge, this is the first study to build a conceptual model to understand and describe the health impacts of PTSD during social unrest through a community-based inclusive development approach. Other than focusing on the problems of the individuals, this model brings also insight into the rights to participation in society. Traditional intervention approach center on improving psychological and physical functioning of the person. Inclusion refers to the respect of rights to participation and self-determination in society. Other than focusing on the problems of the individuals, this model brings also insight into the change required in the social systems and community. Our model emphasized also participation restriction and perceived stigma, which may indicate the necessity of change toward greater inclusion in social systems or even a change in the society itself.

### Strengths and Limitations of This Study

The sample size was adequate for CFA and SEM. Validation of the framework was achieved through the validation of the individual tools and the conceptual model. This framework has the potential to be used in population-based samples as well as in other cultures and contexts. However, this study used mainly an online mode to collect data. Those who did not familiar with the online platform were therefore not be able to participate in this study. Further testing of this model in community health studies on PTSD that involve other cultures, groups, or populations to generate strong evidence are warranted. Besides, we tested mainly the reliability, convergent validity and dimensionality of the four instruments. Further testing on discriminative validity of these instruments is deemed necessary.

## Conclusion

In the present study, a conceptual model describing and evaluating people's health during social unrest was built. This model involved employing a community-based development approach, which is essential to understanding the holistic needs of people during social unrest, to consider individuals' interactions with their social environment. This model can inform practitioners, researchers, and policy makers on the holistic health needs of people in times of social unrest. In particular, it can facilitate the design of programmes for monitoring and improving health and well-being.

## Data Availability Statement

The data contributing to these analyses are held on a secure database and all data generated or analyzed during this study are not publicly available as this may be linked to specific program and staff. Sharing of such data may breach confidentiality. Requests to access the datasets should be directed to chunge@eduhk.hk.

## Ethics Statement

The studies involving human participants were reviewed and approved by the Education University of Hong Kong. The patients/participants provided their written informed consent to participate in this study.

## Author Contributions

EC wrote the main manuscript text and prepared all figures and tables.

## Funding

Data collection of this study was supported by the Additional Research Fund of the Department of Special Education and Counseling at the Education University of Hong Kong.

## Conflict of Interest

The author declares that the research was conducted in the absence of any commercial or financial relationships that could be construed as a potential conflict of interest.

## Publisher's Note

All claims expressed in this article are solely those of the authors and do not necessarily represent those of their affiliated organizations, or those of the publisher, the editors and the reviewers. Any product that may be evaluated in this article, or claim that may be made by its manufacturer, is not guaranteed or endorsed by the publisher.
